# The Paraventricular Nucleus of the Hypothalamus in Control of Blood Pressure and Blood Pressure Variability

**DOI:** 10.3389/fphys.2022.858941

**Published:** 2022-03-16

**Authors:** Bojana Savić, David Murphy, Nina Japundžić-Žigon

**Affiliations:** ^1^Laboratory for Cardiovascular Pharmacology and Toxicology, Faculty of Medicine, Institute of Pharmacology, Clinical Pharmacology and Toxicology, University of Belgrade, Belgrade, Serbia; ^2^Molecular Neuroendocrinology Research Group, Bristol Medical School, Translational Health Sciences, University of Bristol, Bristol, United Kingdom

**Keywords:** blood pressure, blood pressure variability, PVN, vasopressin, oxytocin, baroreflex

## Abstract

The paraventricular nucleus (PVN) is a highly organized structure of the hypothalamus that has a key role in regulating cardiovascular and osmotic homeostasis. Functionally, the PVN is divided into autonomic and neuroendocrine (neurosecretory) compartments, both equally important for maintaining blood pressure (BP) and body fluids in the physiological range. Neurosecretory magnocellular neurons (MCNs) of the PVN are the main source of the hormones vasopressin (VP), responsible for water conservation and hydromineral balance, and oxytocin (OT), involved in parturition and milk ejection during lactation. Further, neurosecretory parvocellular neurons (PCNs) take part in modulation of the hypothalamic–pituitary–adrenal axis and stress responses. Additionally, the PVN takes central place in autonomic adjustment of BP to environmental challenges and contributes to its variability (BPV), underpinning the PVN as an autonomic master controller of cardiovascular function. Autonomic PCNs of the PVN modulate sympathetic outflow toward heart, blood vessels and kidneys. These pre-autonomic neurons send projections to the vasomotor nucleus of rostral ventrolateral medulla and to intermediolateral column of the spinal cord, where postganglionic fibers toward target organs arise. Also, PVN PCNs synapse with NTS neurons which are the end-point of baroreceptor primary afferents, thus, enabling the PVN to modify the function of baroreflex. Neuroendocrine and autonomic parts of the PVN are segregated morphologically but they work in concert when the organism is exposed to environmental challenges *via* somatodendritically released VP and OT by MCNs. The purpose of this overview is to address both neuroendocrine and autonomic PVN roles in BP and BPV regulation.

## Introduction

**Figure 1:**
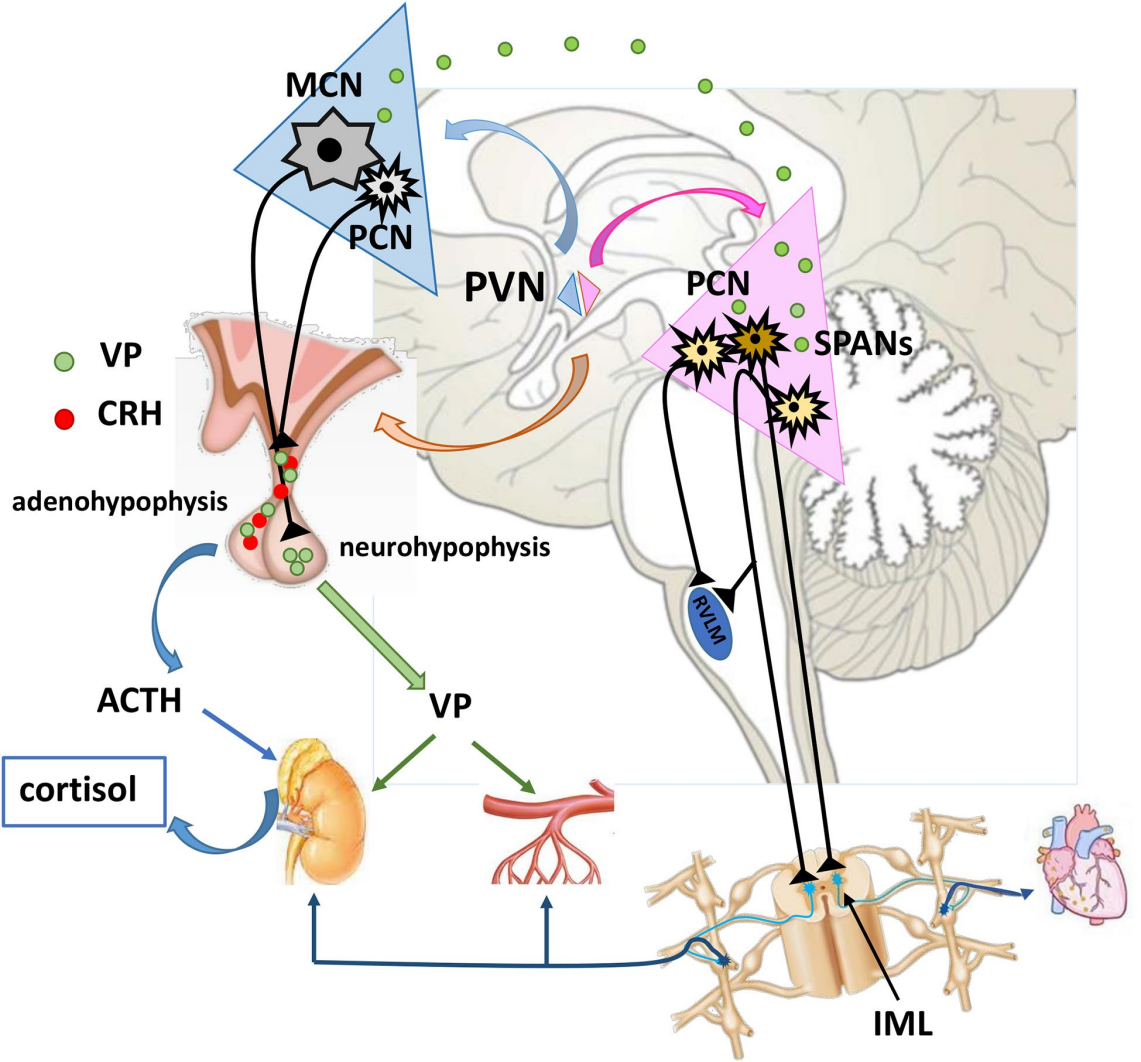
Neuroendocrine and pre-autonomic paraventricular nucleus in cardiovascular regulation. MCNs synthetize VP which is transported *via* their axons to neurohypophysis for systemic release. Once in the circulation, VP reaches distant targets (kidneys, resistance vessels) to exert its effects. Some portion of VP is released intranuclearly, and modulates the activity of pre-autonomic PCNs. These pre-autonomic neurons have the potential to modulate the autonomic outflow toward heart, kidneys and arterioles. Additionally, VP is co-expressed with CRH alternating the reactivity of HPA axis. VP, vasopressin; CRH, corticotropin-releasing hormone; ACTH, adrenocorticotropic hormone; MCN, Magnocellular neuron; PCNs, parvocellular neuron; SPANs, spinally projecting pre-autonimic neurons; RVLM, rostral ventrolateral medulla; IML, intermediolateral nucleus.

Occupying a small portion of the vertebrate brain (1%), the PVN is a highly organized effector structure (Swanson, 1995; Benarroch, 2005). This hypothalamic nucleus is located bilaterally around the third ventricle (Badoer, 2001). Morphological studies of the PVN reveal different cell populations within its borders, such that the PVN can be divided into at least three magnocellular (anterior, posterior and medial subnuclei) and five parvocellular (dorsal, lateral, medial, periventricular and anterior subnucleus) compartments (Swanson and Kuypers, 1980; Sawchenko and Swanson, 1982a; Swanson and Sawchenko, 1983; Badoer, 2001; Pyner, 2009). Two functionally separate areas of the PVN, neuroendocrine and autonomic, subserve its potential to regulate BP, making the PVN a major integrative site of cardiovascular function (Swanson and Kuypers, 1980; Swanson and Sawchenko, 1983; Son et al., 2013; Sladek et al., 2015). Blood pressure, which is defined by peripheral vascular resistance and cardiac output (resultant of heart rate and stroke volume), is modified by both neuroendocrine and autonomic premotor PVN in at least three different effector pathways (Figure 1): neurosecretory magnocellular, neurosecretory parvocellular, and pre-autonomic parvocellular neural pathway (Badoer, 2001; Sladek et al., 2015).

## Modulation of Blood Pressure by the Neuroendocrine PVN

Over 30 distinct neurotransmitters and neuromodulators have been identified to be synthesized within the PVN (Swanson and Sawchenko, 1983). Most abundantly expressed are vasopressin (VP) and oxytocin (OT), which are produced by the magnocellular neurons (MCNs) of the PVN (Swanson and Sawchenko, 1980; Sladek et al., 2015). VP is best known for its role in maintaining cardiovascular and body fluid balance, whereas OT takes part in parturition, lactation and accompanying reproductive behaviors (Gimpl and Fahrenholz, 2001; Koshimizu et al., 2006; Stoop, 2014). Additionally, VP and corticotropin-releasing hormone (CRH) expressing parvocellular neurons (PCNs) of the PVN possess a secretory capacity and mediate the central response of the hypothalamic–pituitary–adrenal (HPA) axis to stress (Swanson and Sawchenko, 1980; Sawchenko et al., 1996; Benarroch, 2005; Sladek et al., 2015). Magnocellular and parvocellular neuroendocrine neurons of the PVN initiate a downstream chain of events that dictate changes in BP.

### The Role of Neurosecretory MCNs in BP Regulation

Magnocellular neurons express VP and OT in large quantities (Dierickx, 1980). Located mostly in the medial and the lateral posterior subdivision of the PVN, MCNs send their axonal projections to neurohypophysis, from where VP and OT is released into the systemic circulation (Armstrong et al., 1980; Swanson and Sawchenko, 1983; Sladek et al., 2015). Once secreted into blood, these peptide hormones act upon distant targets (Dierickx, 1980; Gutkowska et al., 2000; Japundžić-Žigon, 2013). VP and OT exert their effects through activation of cognate receptors that are expressed both in the brain and periphery (Brinton et al., 1984; Ostrowski et al., 1992; Hirasawa et al., 1994; Adan et al., 1995; Kato et al., 1995). VP receptors (VR) and OT receptors (OTR) are a subfamily of G coupled receptors (G protein-coupled receptors—GPCRs) with high structural homology (85% homology found between V1aR and OTR; Barberis et al., 1998; Thibonnier et al., 2002; Koshimizu et al., 2012). In the periphery, VP mainly engages V1aR and V2R, while in the central nervous system VP action is mostly mediated by V1aR and far less by V1bRs (Ostrowski et al., 1992, 1994; Young et al., 2006; Roper et al., 2011; Russell and Brunton, 2017).

#### Peripheral VP and OT in Blood Pressure Regulation

The strongest stimulus for the MCNs to secret VP into the bloodstream is hyperosmotic change (McKinley et al., 2004). Hyperosmolality of blood is sensed by circumventricular organs in the periventricular region of the third ventricle (anteroventral third ventricle—AV3V), which is devoid of the blood brain barrier. The circumventricular subfornical organ (SFO) and *organum vasculosum laminae terminalis* (OVLT) are richly vascularised, and their neurons easily sense perturbations in blood osmolality (Leng et al., 1989; Cunningham and Sawchenko, 1991; McKinley et al., 2004). From these structures, direct or indirect (*via* medial preoptic nucleus—MnPO) excitatory axonal projections to MCNs, stimulate the secretion of VP into circulation (Leng et al., 2001; McKinley et al., 2004). Additionally, several other stimuli trigger VP secretion: decreased blood volume and pressure which induce reduction of stretch in low-pressure receptors of venous system and high-pressure baroreceptors respectively and ANG II (Japundžić-Žigon et al., 2020).

The best known roles of peripheral VP are water conservation in kidneys and vasoconstriction (Altura and Altura, 1984; Bankir, 2001; Sladek et al., 2015). Indeed, VP is often referred to as antidiuretic hormone (ADH), due to its role in water conservation by the activation of V2Rs in the kidneys (Ostrowski et al., 1993; Gordan et al., 2015). Even small changes in VP concentration in the serum will activate renal V2Rs, with highest affinity to VP, to preserve water (Japundžić-Žigon, 2013). V2Rs are located in the basolateral membrane of the epithelial cells of the collecting ducts of the kidney. Activation of V2R is responsible for a cascade of events that involve phosphorylation of aquaporin 2 (AQP-2) and its translocation into the luminal membrane of epithelial cells, leading to reabsorption of water by the kidneys. VP also affects AQP-2 transcription rates and increases AQP-2 protein abundance (Imbert et al., 1975; Knepper, 1997; Bankir, 2001; Wilson et al., 2013; Jung and Kwon, 2016).

The vasoconstrictor effect of VP is mediated by V1aRs located on blood vessels. Although *in vitro* studies confirm VP as a most potent vasoconstrictor agent, relatively high concentrations of VP are necessary to elevate BP *in vivo* under basal physiological conditions (Altura and Altura, 1984; Johnston, 1985; Koshimizu et al., 2006) in respect to much less potent vasoconstrictors at molar level such as angiotensin II and noradrenalin. Nevertheless, vascular V1aRs are crucial for BP maintenance during hypovolemia and hypotension (Koshimizu et al., 2012). Also, in some vascular beds, such as in lung, liver and kidneys VP activates V1aR and V2R to produce nitrogen monoxide (NO) dependent vasodilatation (Liard, 1984; Hirsch et al., 1989; Russ and Walker, 1992; Aki et al., 1994; Koshimizu et al., 2006).

Circulating VP can circumvent the blood brain barrier and reach centrally located receptors (Landgraf and Neumann, 2004). The most prominent impact of peripheral VP on blood pressure is exerted at the level of area postrema (AP), where it can modify the activity of the baroreflex (Brizzee and Walker, 1990; Hasser et al., 1997). Even though the VP effects on baroreflex are controversial, the majority of studies report enhancement of baroreflex sensitivity (BRS; Brizzee and Walker, 1990; Hasser and Bishop, 1990; Hasser et al., 1997; Koshimizu et al., 2006). Further support is provided by experiments which show that lesions of the AP region disable peripheral VP to modulate baroreflex (Hasser et al., 1997). Also, VP deficient Brattleboro rats exhibit decreased BRS (Imai et al., 1983b). Pharmacological studies suggest that this effect is conveyed by V1aR (Imai et al., 1983a; Hasser and Bishop, 1990; Sampey et al., 1999). The effect of VP on BRS is complex, and when VP is released centrally during stress and exercise, it will act oppositely and reduce BRS *via* medullary V1aRs (Unger et al., 1986).

Afferents that originate from stretch receptors and baroreceptors are tonically active under physiological conditions and inhibit VP secretion from MCNs, while decrease in blood volume and BP leads to disinhibition and consequent release of VP (Bisset and Chowdrey, 1988). Before it reaches the PVN, the information from the baroreceptors is conveyed *via* the *nucleus tractus solitarius* (NTS) and ventrolateral medulla (VLM). Afferents arising from the NTS (A2 type neurons) and VLM (A1 cell group) that project to MCNs are primarily noradrenergic (Sawchenko and Swanson, 1982b). Putative inhibitory mechanisms involve NTS residing GABAergic neurons which silence the noradrenergic excitatory pathways directed to the magnocellular subdivision of the PVN (Jhamandas and Renaud, 1987; Bisset and Chowdrey, 1988; Leng et al., 1999).

Altogether, it seems that the minor effect of VP on BP maintenance under basal physiological conditions is due to the BRS enhancement *via* AP and consequent decrease in heart rate which efficiently oppose its vasoconstrictor performance on the periphery (Abboud et al., 1990; Koshimizu et al., 2006).

Oxytocin is a nonapeptide hormone best known for inducing uterine contractions during labor and milk ejection during lactation (Dale, 1906; Ott and Scott, 1910; Du Vigneaud, 1954). Apart from these primary roles, OT is involved in a large number of physiological activities, including cardiovascular control (Maier et al., 1998). OT secretion in hypothalamus is not only sex dependent, and it can be modulated by hyperosmotic stimuli, hypovolemia and ANG II (Kadekaro et al., 1992; Gimpl and Fahrenholz, 2001). Mechanisms underlying OT involvement in BP regulation are not yet fully elucidated (Gutkowska et al., 2000). OT activity usually correlates with BP decrease in many species. Experiments with RNA interference report that blocking brain OT RNA leads to an increase in BP (Petersson et al., 1996, 1997; Maier et al., 1998). One of the putative mechanisms behind OT modulation of BP lies in its engagement with electrolyte excretion in the kidneys. It is suggested that OT induced natriuresis is mediated by atrial natriuretic peptide (ANP; Verbalis et al., 1991; Conrad et al., 1993; Gutkowska et al., 2000). However, this natriuretic effect is not confirmed in humans, and it appears that it is present only in certain species, such as rats (Rasmussen et al., 2004).

Since VP and OT share very similar primary structures (only differing by 2 amino acids at positions 3 and 8), as well as their receptors, OT can bind with high affinity to V1aRs as well as OTRs (Gimpl and Fahrenholz, 2001; De Bree et al., 2003). OT receptors have a wide distribution in the body (Adan et al., 1995). As well as the reproductive system, OTRs can be found within the brain and heart (Adan et al., 1995; Gutkowska et al., 1997). OT induced peripheral vasoconstriction can be mediated by V1aRs. *In vitro* studies reveal that the potency of OT to contract blood vessel smooth muscle cells is much less than VP, with the exception of the umbilical artery at term (Altemus et al., 2001). When vascular tone is already increased, OT will produce NO dependent vasodilatation in some vascular beds, like in basilar arteries (Katusic et al., 1986; Russ et al., 1992; Thibonnier et al., 1999). It seems that OT is not responsible for modulating the peripheral resistance in pregnant rats, and does not play a significant role in setting the levels of BP under physiological conditions (Miller et al., 2002).

Even though experimental evidence does not support strong involvement of VP and OT in BP regulation under baseline physiological conditions, studies with VP and OT gene knock-out mice provide a contrasting insight. VP knock-out mice exhibit lower basal values of BP, while OT deficient mice demonstrate elevated BP and HR values in comparison to wild type controls (Bernatova et al., 2004; Koshimizu et al., 2006). Apart from being normotensive, VP deficient Brattleboro rats exhibit decreased BRS. However, it should be noted that the complex and unpredictable developmental compensations occurring in global knock-out mice makes the interpretation of adult phenotypes problematic (Valtin, 1982; Imai et al., 1983b; Bohus and de Wied, 1998).

#### Intranuclear OT and VP in Blood Pressure Regulation

Vasopressin and OT can be synthesized and released from dendrites and soma of MCNs and this can happen without cell depolarization (Scala-Guenot et al., 1987; Moos et al., 1989, 1998; Pow and Morris, 1989; Neumann et al., 1993b; Ludwig et al., 1995, 2002; Hurbin et al., 1998, 2002). VP in the extracellular space can exert both autocrine and paracrine effects (Ludwig and Stern, 2015). Intranuclear VP can activate V1aRs and V1bR expressed on MCNs, or it can spill over through the extracellular space into the cerebrospinal fluid (Landgraf and Neumann, 2004) and reach remote targets (Hurbin et al., 1998, 2002; Son et al., 2013). It has been proposed that intranuclear receptors optimize the firing rate of the entire population of MCNs to best respond to physiological demands (Son et al., 2013). Somatodendritic release depends on the quality and intensity of stimulus and it can be regulated independently from systemic release (Pow and Morris, 1989; Neumann et al., 1993a; Ota et al., 1994; Kendrick et al., 1997; Gouzènes et al., 1998; Landgraf and Neumann, 2004). It has been suggested that the autocontrol of MCN by intranuclear VP can be either inhibitory or excitatory (Inenaga and Yamashita, 1986; Gouzènes et al., 1998; Dayanithi et al., 2000; Landgraf and Neumann, 2004). Additionally, somatodendritically released VP (Figure 1) can activate surrounding silent MCNs (Moos et al., 1998), or depolarize neighboring interneurons and PCNs (Carette and Poulain, 1989; Son et al., 2013). Activation of autonomic PCNs through engagement of V1aRs, especially during hyperosmotic stimulus, is particularly important in terms of integration of neuroendocrine and autonomic regulation of blood pressure (Swanson and Sawchenko, 1980; Son et al., 2013). It has also been reported that intranuclear V1bRs participate in setting sympathetic outflow toward the kidneys (El-Werfali et al., 2015).

### The Role of PVN Neurosecretory PCNs in BP Regulation

Stress is an attributed risk factor for cardiovascular diseases that can trigger bad clinical outcomes (Hjemdahl, 2002; Rosmond, 2005). PVN involvement in the stress response has been documented in spontaneously hypertensive rats (SHRs) and other experimental models (Benarroch, 2005). The PVN promotes several aspects of hemodynamic regulation during stress (Herman et al., 1996; Benarroch, 2005). Anterior and medial PCNs that synthesize CRH, are responsible for activating the HPA axis during exposure to stress (Rivier and Vale, 1983; Swanson et al., 1983; Sawchenko et al., 1996; Sladek et al., 2015). Apart from expressing CRH, PCNs produce VP as secretagogue. It appears that the CRH:VP ratio is dictated by the type of stressor, and is crucial for maintaining responsiveness of the HPA axis during chronic stress (Sawchenko and Swanson, 1982a; Swanson and Sawchenko, 1983; Whitnall et al., 1985; de Goeij et al., 1992; Aguilera, 1994; Sawchenko et al., 1996; Amaya et al., 2001). Both CRH and VP are axonally transported to eminentia mediana and released into the portal circulation. Borne by the portal bloodstream, they reach adenohypophysis, where they act on corticotropic cells. CRH stimulates the release of adrenocorticotropic hormone (ACTH), whereas VP potentiates its release by activating V1bRs (Benarroch, 2005). Once in the systemic circulations (Figure 1), ACTH acts on the cells of *zona fasciculata* in adrenal gland to release glucocorticoids (Myers et al., 2012; Sladek et al., 2015). Cortisol when in excess, has been shown to contribute to hypertension (Kelly et al., 1998). Additionally, elegant ontogenetic experiments suggest that CRH PCNs can increase BP and heart rate *via* axonal projections to the NTS (Wang et al., 2019), involving corticotropin-releasing hormone receptor type 2 (CRHR2), also associated with hypertension triggered by intermittent hypoxia (Wang et al., 2019).

It has been shown that forced swimming and social confrontation, stressors employed in experimental conditions, can induce somatodendritic release of VP and OT (Wotjak et al., 1996; Engelmann et al., 2001; Ebner et al., 2005), which in turn modifies the activity of the HPA axis. Intranuclear VP has an inhibitory effect on CRH PCNs and consequently reduces secretion of ACTH (Wotjak et al., 1996, 2002; Bosch et al., 2004; Ebner et al., 2005). Extracellular OT exhibits both inhibitory and excitatory influence on the activity of the HPA axis (Neumann et al., 2000; Heinrichs et al., 2002; Neumann, 2002; Landgraf and Neumann, 2004).

## The Role of the “Pre-Autonomic” PVN in BP Regulation

Apart from being locally regulated, the cardiovascular system is subject to central control by numerous relevant brain areas (Dampney, 1994). It is well established that PVN takes an important central place in such control. Numerous studies show that the PVN is implicated in the heightened sympathetic tone observed in hypertension (Allen, 2002; Dampney et al., 2018). The autonomic PVN consists of morphologically and functionally diverse cell populations with a specific topography (Stern, 2001; Dampney et al., 2018). It occupies ventromedial, lateral and dorsal (dorsal cap) subdivision of PVN (Nunn et al., 2011; Sladek et al., 2015). More precisely, these PCNs are pre-autonomic, since they control functionally different sympathetic and parasympathetic centers downstream in the medulla: NTS, dorsal motor nucleus of vagus (DMV) and rostral ventrolateral medulla (RVLM), and the spinal cord: intermediolateral nucleus (IML). Therefore, the “pre-autonomic” premotor PVN is responsible for altering the autonomic output toward the cardiovascular and renal systems (Strack et al., 1989a; Coote et al., 1998; Portillo et al., 1998; Coote, 2005; Dampney et al., 2018). Hence it is not surprising that PVN is commonly referred to as an “autonomic master controller,” a term originally introduced by Loewy (1991). There are at least 3 pathways (Figure 1) through which PVN modulates sympathetic outflow (Pyner, 2009):

The first pathway includes PVN pre-sympathetic neurons that have axonal projections that terminate on somata of motor sympathetic preganglionic neurons (SPNs) in the thoraco-lumbal IML. These neurons are usually named as spinally projecting pre-autonomic neurons (SPANs; Dampney, 1994; Badoer, 2001; Nunn et al., 2011). The IML is the final spot of central integration and origin of preganglionic fibers which regulate the activity of blood vessels, heart, kidneys and adrenal gland. Therefore, this is a particularly important target of the PVN pre-autonomic neurons, displaying its enormous potential to directly alternate neurogenic output to the cardiorenal system (Strack et al., 1989a; Dampney, 1994; Badoer, 2001).

The second pathway involves pre-sympathetic PVN neurons that exert indirect influence on sympathetic activity. These neurons terminate at the level of the motor pressor nucleus of the RVLM, responsible for setting sympathetic tone. From there, second order neurons arise and project to SPNs in the thoracic and lumbar IML and change sympathetic outflow toward the cardiovascular system and the kidneys (Dampney, 1994; Badoer, 2001). In general, PVN neurons projecting to RVLM are in greater number than PVN neurons projecting monosinaptically to IML. Therfore, RVLM is a structure embedding most of the pre-sympathetic neurons, which are projecting to IML to exert major autonomic cardiovascular control (Badoer, 2001). Some of the sympatho-excitatory effects in the PVN-RVLM pathways are glutamate mediated (Yang and Coote, 1998). Additionally, evidence has emerged, that PVN can interfere with this PVN-RVLM pathway through its glutamatergic synapses within medial subnucleus of the NTS (mNTS; Kawabe et al., 2008). Using anterograde and retrograde tracing, Kawabe working group confirmed the presence of such bilateral glutamatergic projections spanning to mNTS. They showed that unilateral PVN stimulation with N-methyl-D-aspartic acid (NMDA) leads to increase in mean arterial pressure and greater splanchic nerve activity, and that this effect is emphasized by bilateral blockade of glutamate ionotropic receptors within the mNTS. The authors suggested that PVN glutamatergic pathways directed toward NTS, stimulate inhibition of RVLM-mediated cardiovascular overactivity. Therefore, PVN stimulation with NMDA evokes both RVLM and NTS neuronal routes, but the effects are opposing (Kawabe et al., 2008). Further, same authors showed that tachycardia induced by NMDA stimulation of the PVN is a result of conjoined inhibition of vagal (*via* ionotropic glutamate and GABA receptors in the mNTS) and activation of sympathetic outputs (*via* spinal ionotropic glutamate) toward heart, without involvement of spinal VRs and OTRs (Kawabe et al., 2009).

A third pathway is represented by PVN pre-autonomic neurons that can change sympathetic tone both directly and indirectly. Around 30 % of PVN neurons innervate SPNs in the IML, but send collaterals to the RVLM, thus having a dual regulatory role (Badoer, 2001).

The majority of PVN autonomic regulatory function is conveyed by SPANs (Coote, 2007). Since the relevant portion of SPANs is implicated in cardiovascular control (Badoer, 1996; Badoer, 2001; Pyner, 2009; Nunn et al., 2011), this makes them an attractive target for new drug development.

Although their exact functions have yet to be elucidated, SPANs have been suggested to regulate blood volume (Lovick et al., 1993; Pyner and Coote, 2000), circadian variations in BP (Cui et al., 2001), stress induced cardiovascular responses (Jansen et al., 1995a) and much more (Nunn et al., 2011). For these reasons SPANs are referred to as “central command neurons” (Jansen et al., 1995a). Discovering their primary physiological function is further complicated by the fact that SPANs express a lot of different neuroactive substances/neurotransmitters. Indeed, it appears that the majority of these neurons synthesize more than one neurotransmitter (Nunn et al., 2011). The largest portion of SPANs (up to 40 %) is positive for VP and OT (usually co-expressed), as well as dynorphin (Hallbeck and Blomqvist, 1999; Xi et al., 1999; Hallbeck, 2000; Hallbeck et al., 2001). Others possess met-endorphin (up to 20%) and dopamine, met-enkephalin (up to 10 %), leu-enkephalin, somatostatin, ANG II and ANP (Sawchenko and Swanson, 1982a; Cechetto and Saper, 1988; Strack et al., 1989b; Jansen et al., 1995b).

Functional *in vivo* studies show that spinal levels of VP and OT increase with PVN stimulation (Pittman et al., 1984; Malpas and Coote, 1994). The presence of V1aR and OTR has been confirmed in the gray matter of the spinal cord (Desaulles et al., 1995; Sermasi et al., 1998; Pyner, 2009). Intrathecal pretreatment with V1aR antagonist in the lower thoracic region prevents the increase in renal sympathetic nerve activity and mean arterial pressure normally triggered by the stimulation of the PVN (Malpas and Coote, 1994). It cannot be excluded that some of the OT effects are conveyed *via* spinal V1aRs as well (Sermasi and Coote, 1994). Similar effects are also observed with OTR selective antagonists which abolish the effects of increased heart rate following the PVN stimulation (Yang et al., 2009). These experimental data highlight VP and OT as PVN SPAN transmitters. VP and OT induce cardio-acceleratory and pressor effects in lower and upper thoracic spinal cord respectively (Pyner, 2009). The majority of vasopressinergic projections implicated in cardiovascular control originate in the lateral and ventral medial subdivision of parvocellular PVN and some portion is found in dorsal parvocellular part (Riphagen and Pittman, 1989; Hallbeck and Blomqvist, 1999). Oxytocinergic SPANs are located in the lateral parvocellular region and dorsal cap (Sawchenko and Swanson, 1982a; Pyner, 2009).

The effects of dopamine as a putative SPAN neurotransmitter are controversial (Strack et al., 1989b; Nunn et al., 2011). Some studies on rats suggest its excitatory influence on SPNs, while others report an inhibitory activity (Gladwell et al., 1999; Gladwell and Coote, 1999a,b; Yang et al., 2002).

Pharmacological experiments with a glutamate antagonist, following chemical stimulation of PVN, imply glutamate as an additional excitatory neurotransmitter of SPANs (Yang et al., 2002).

Although SPANs are positive for enkephalins and ANG II, their influence on sympathetic outflow has not yet been recorded (Sawchenko and Swanson, 1982a; Cechetto and Saper, 1988; Jansen et al., 1995b; Hallbeck and Blomqvist, 1999; Hallbeck et al., 2001). Despite its abundant expression in the nervous system, the presence of inhibitory γ-aminobutyric acid (GABA) as a SPAN output neurotransmitter has not been confirmed (Macdonald and Olsen, 1994; Watkins et al., 2009).

### Modulation of SPAN Activity

Spinally projecting pre-autonomic neuron activity can be modulated in various ways. A lot has been discovered about the neurotransmitter content of SPANs, but the data regarding receptors expressed on SPANs and molecules their receptors bind is lacking (Nunn et al., 2011). Learning about the neurotransmitters which can modulate the activity of SPANs will open venues to novel therapeutic agents.

It is well established that the PVN is involved in increased sympathetic activity driven by osmotic stimulation, but the mechanisms behind it are poorly understood. Some studies suggest involvement of intranuclear VP released from soma and dendrites of MCNs (Son et al., 2013; Ribeiro et al., 2015). Intranuclear VP stimulates V1aRs expressed on pre-sympathetic parvocellular subdivisions (including SPANs) of the PVN. This leads to increase in sympathetic outflow toward heart, blood vessels and kidneys, followed by BP increase, suggesting this pathway as potential pathophysiological mechanism in neurogenic hypertension (Son et al., 2013; Ribeiro et al., 2015).

Under basal physiological conditions, PVN neurons are tonically inhibited by surrounding GABA neurons, keeping the spontaneously generated nerve impulses at low rate, despite the excitatory influence of glutamate (Kannan et al., 1989; Martin et al., 1991; Badoer et al., 2002; Li et al., 2006). SPANs are confirmed to be silenced by GABA (Cui et al., 2001; Li et al., 2002). This inhibition could be dependent on extrasynaptic GABA (“volume” GABA transmission), but it can also be affected by the rate that glial cells take up GABA from the extracellular space (Brickley et al., 1996; Farrant and Nusser, 2005; Park et al., 2009). GABAA α2-subunit is abundantly expressed in the PVN (Fritschy and Mohler, 1995). Blockage of GABAA receptors by the selective antagonist bicuculline leads to an increase in BP and heart rate (Martin et al., 1991; Martin and Haywood, 1993). Interfering with GABA orchestrated SPAN activity is an appealing therapeutic opportunity, since increasing GABA inhibitory influence would lead to reduction of sympathetic tone and consequently BP decrease (Nunn et al., 2011).

Although injections of ATII in the PVN change blood pressure *via* angiotensin II receptor type 1 (AT1R; Bains et al., 1992), and this involves the activity of SPANs, it seems that this connection is indirect (Bains and Ferguson, 1995; Li et al., 2003). AT1Rs are expressed in parvocellular division of the PVN on neurons projecting to medulla, not IML (Oldfield et al., 2001; Cato and Toney, 2005).

Spinally projecting pre-autonomic neurons, as well as PCNs which project to RVLM are barosensitive. Under basal physiological conditions, these neurons exhibit spontaneous activity, but they are inhibited by rising pressure (Dampney et al., 2018). Axons arising from caudal NTS neurons terminate on PCNs in the dorsal cap of the PVN. Putative targets of these projections are pre-sympathetic PCNs or GABA interneurons (Pyner, 2009; Dampney, 2017). Other studies do not impose an important role of SPANs in the baroreflex response (Haselton et al., 1994). Volume load is another feed-back mechanism that can modify the activity of SPANs, with PVN being a command center of low-pressure blood volume receptors located in the veno-atrial junction (Gupta et al., 1966; Lovick and Coote, 1988; Lovick et al., 1993; Deng and Kaufman, 1995). Additionally, SPANs can be modulated by some types of stressors, such as psychological stress. It has been shown that conditional fear engages around 10 % of SPANs (Carrive and Gorissen, 2008; Dampney et al., 2018). Also SPANs are affected by temperature, different humoral factors, and inputs from higher brain areas (Dampney et al., 2018).

## Paraventricular Nucleus and Blood Pressure Short-Term Variability

The peripheral sympathetic nervous system controlling the cardiovascular system has rhythmic activity that creates distinct patterns of sympathetic nerve discharge (SND) in response to physiological demands and pathophysiological conditions. Using frequency analysis of SND, components of SND were identified including fast cardiac and respiratory rhythms, and slow vasomotor rhythm. It is generally believed that the brain is the source of SND. Thus, which parts of the brain are involved, and how they generate peripheral SND rhythms is still unanswered. Two main theories have been postulated, both of which raised considerable criticism. A theory of a central oscillator/pacemaker suggests that the RVLM is the main structure responsible for generating peripheral sympathetic discharge patterns *via* axonal projections to preganglionic neurons in IML. Recordings of intracellular neuronal activity from medullary slices, uncovered ramp like depolarization following each action potential, leading to subsequent action potential suggestive of pacemaker activity (Li and Guyenet, 1996). This observation was seriously challenged by the *in vivo* study in rats by Lipski et al. (1996) who observed that RVLM neurons fire irregularly, at much higher discharge rates than the ones in SND, with no evidence of gradual depolarization between individual action potentials (Lipski et al., 1996). They suggested that the regular pattern of firing of RVLM neurons seen in medullary slices, was produced by deafferentation. The work of Lipski and colleagues imposes the network hypothesis, where the activity of pre-sympathetic neurons depends on their antecedent excitatory inputs opposed by tonic inhibitory inputs (setting the level of their excitability). For detailed review refer to Malpas (1998). Geber and Barman postulated a theory of a network of brainstem neurons, whose combined action creates inherent rhythmicity entrained by the baroreflex. These include sympatho-excitatory and sympatho-inhibitory neurons distributed over a wide portion of the lower brainstem that do not have necessarily spontaneous activity (Barman and Gebber, 2000).

In addition to RVLM neurons, hypothalamic PVN SPANs provide monosynaptic inputs to preganglionic neurons in IML. Malpas and Coote (1994) were first to provide evidence in anesthetized rats that chemical stimulation of the PVN by microinjections of homocysteic acid increases the amplitude of renal SND, by recruiting more active fibers, and that this can be blocked by intrathecal injection of V1R antagonist. At the same time the frequency of renal SND, which is modulated by periodic baroreflex inputs (Ninomiya et al., 1990; Malpas and Ninomiya, 1992), remained unaffected. The first experimental evidence that PVN SPANs can incite BPV at the same stimulation frequency was demonstrated in rats by Stauss and associates (Stauss and Kregel, 1996; Stauss et al., 1997). PVN electrically stimulated at frequencies between 0.1 Hz - 0.5 Hz were found to create the same frequencies in the SND pattern and generate BPV in the low frequency (LF) band, which is abolished by α-adrenergic blockade (Japundzic et al., 1990). Stimulation frequencies above 0.5 Hz did not induce BP variations as the blood vessels behaved like cut off filters to high frequencies. Studies on isolated rat vascular smooth muscle cells showed that transmission of fast SND rhythms to BPV is limited by the sluggish, metabotropic α-adrenoceptor signaling, and not by an intrinsic inability of the cells to contract and relax at higher rates (Julien et al., 2001). Thus, SND frequencies higher than 0.5 Hz in rats induce vasoconstriction, increase peripheral resistance and the mean value of BP. It follows that the faster components of BPV, the cardiac component and the respiratory component, are non-neural, created by the perturbations of the circulation induced by the contracting heart and inspiratory movements (Japundzic et al., 1990; Japundzic-Zigon, 1998). The non-neural origin also stands for the slowest, and dominant component of BPV, the very low frequency (VLF) component, which was found to be created by inherent myogenic activity of mesenteric and renal vasculature (VanBavel et al., 1991; Janssen et al., 1995). Although non-neuronal in nature, all the components of BPV can be modulated by the activity of the nervous system and neurohormones (Japundžić-Žigon et al., 2020).

We have investigated the neurochemical contribution of the PVN to short-term BPV, and found that both VP and OT modulate short-term BPV. Using pharmacological and genetic tools in conscious rats we have shown that VP modulates BPV in a complex manner: peripherally as a hormone and centrally as neurotransmitter/modulator (Japundzic-Zigon, 2001, 2013; Japundzić-Zigon et al., 2004, 2018, 2020; Milutinović et al., 2006a,b; Stojicić et al., 2008; Milutinović-Smiljanić et al., 2013; Lozić et al., 2016; Savić et al., 2020). Using spectral analysis of BPV, we found that peripheral administration of non-peptide and selective V1aR or V2R antagonists to conscious normotensive rats under baseline physiological conditions increases BPV, suggesting a buffering role for VP in the VLF domain (Japundzic-Zigon, 2001). We postulated that the decrease of VLF-BPV by VP could be mediated either by the enhancement of the baroreflex sensitivity which normally opposes VLF-BPV, or by the modulation of vasomotion in mesenteric and renal vascular beds (Janssen et al., 1995). In SHR, the buffering capacity of VP on BPV under baseline condition is not preserved (Japundzić-Zigon et al., 2004). This could be due to pathological remodeling of the vasculature in SHR (Head, 1991; Vågnes et al., 2000) making it more sensitive to vasoconstrictors, including VP. Another possibility, even more likely is that impaired baroreflex in SHRs (Dampney, 2017) reduces its capacity to buffer VLF-BPV (Dampney, 2017). However, during hemorrhage, when VP is released in excess in blood, in support of circulation, it acts similarly in normotensive and hypertensive rat strains, and prevents the respiratory related high frequency (HF-BPV) increase, possibly as a consequence of V1aR-mediated vasoconstriction which prevents the unloading of thoracic vessels underlying HF-BPV increase.

Intracerebroventricular injection of selective V1aR, V1bR and V2R antagonists to conscious normotensive rats uncovered that VP acts also centrally to buffer VLF-BPV under baseline physiological conditions by the stimulation of V1aRs possibly in AP, accessible from both sides of the blood brain barrier. However, when VP release is stimulated by stress (Stojicić et al., 2006, 2008; Milutinović et al., 2006b), or by drugs (Milutinović et al., 2006a) and when VP is injected centrally (Milutinović et al., 2006b), an increase of the sympathetically mediated LF-BPV and of the respiration mediated HF-BPV was observed (Milutinović et al., 2006a,b). These effects of VP could involve central V1aR found in abundance in the RVLM where integration of the sympathetic outflow to vasculature occurs; and in the pre-Bötzinger area, where the breathing pattern is set. These central effects of VP could be beneficial and contribute to the lifesaving effect of VP in hemorrhagic, septic and cardiogenic shock as a results of increased tissue oxygenation and additional sympathetic activation, acting synergistically with powerful V1aR mediated peripheral vasoconstriction (Levy et al., 2018). VP can also modulate respiration indirectly (Stojicić et al., 2008) by an anxiogenic action characterized by hyperventilation that occurs possibly by the stimulation of V1bR in bed nucleus *stria terminalis* (Griebel et al., 2002).

Paraventricular nucleus is a recognized key integrative site of the behavioral, autonomic and endocrine response to stress, expressing V1aRs and V1bRs on somata and dendrites of MCNs and surrounding glia. Stress has been shown to induce VP and OT release in the PVN too (Nishioka et al., 1998; Landgraf and Neumann, 2004), and we have shown that VP and OT act locally, in an autocrine and paracrine manner, to modulate the neuro-cardiogenic stress response. Using adenoviral gene transfer technology we have increased the gene expression and the number of V1aRs in the PVN of Wistar rats. The V1aR rat phenotype had decreased sensitivity of the baroreflex under baseline physiological conditions which was further decreased by stress along with a marked increase of the sympathetically mediated LF-BPV and LF heart rate variability (LF-HRV). These effects could be abolished by intranuclear application of V1aR antagonist. This suggests that V1aR in the PVN can increase the sympathetic outflow to the periphery and modulate BPV and HRV during stress (Lozić et al., 2014; Japundžić-Žigon et al., 2020). In clinical practice, the increase in LF-HRV has been found to predict the occurrence of life threatening arrhythmias in susceptible populations (Huikuri and Stein, 2013). In contrast to V1aR, OTR over-expression in the PVN of Wistar rats had no effect on BPV under baseline conditions but this rat phenotype exhibited reduced baroreflex desensitization by stress and reduced LF-BP increase suggesting that OTR over-expressing rat phenotype is resilient to stress (Lozić et al., 2014; Japundžić-Žigon et al., 2020). In this context, it is important to stress that the modulation of the baroreceptor desensitization during stress by VP seems to be complex and involves more than one central structure and type of VRs (Milutinović-Smiljanić et al., 2013).

A number of clinical studies in hypertensive patients unequivocally show that enhanced BPV increases the risk for developing cardiovascular complications (Mancia and Grassi, 2000). Thus, BPV, and especially sympathetically derived LF-BPV, emerged as an independent predictor of stroke, coronary artery disease, heart and renal failure, as well as all-cause mortality (Mancia et al., 1994; Mancia and Parati, 2003; Messerli et al., 2019; Parati et al., 2020). Our group investigated expression of PVN VP and VRs in the genesis of hypertension. Borderline hypertensive rats (BHR) have a genetic predisposition for hypertension and will develop it when exposed to environmental challenges. Under baseline physiological conditions BHRs have increased expression of VP and V1bR in the PVN and consequently increased plasma VP concentrations, as a constitutive trait (Savić et al., 2020). Spectral markers of sympathetic activity toward blood vessels, LF-BP, and the heart, LF/HF-HR, are comparable to normotensive rats under baseline physiological conditions, suggesting that increased expression of V1bR and VP in BHRs is confined to magnocellular (endocrine) portion of the PVN affecting plasma VP only. However, when exposed to repeated stress and prolonged isotonic saline load, BHRs exhibited LF-BPV increase depicting sympathetic overload, and overt hypertension. In these rats no changes in VP and VR gene transcription in the PVN was noted. Moreover, systemic VP release was decreased, refuting involvement of VP in stress-induced hypertension (Savić et al., 2020).

## Conclusion

It is well established that PVN has a paramount role in cardiovascular regulation and contributes to the severity of cardiovascular diseases. Both neuroendocrine and autonomic PVN, have a dynamic part in adjusting the circulation to physiological demands and in the modulation of short-term BPV. Thus, elucidating the tightly intertwined mechanisms underlying complexity of the PVN network in health and disease may open up new therapeutic venues.

## Author Contributions

BS, DM, and NJ-Ž: outlining paper draft, writing and refining the manuscript, and critical reading of the manuscript. All of the authors have read and approved the manuscript.

## Funding

This work was supported by Serbian Ministry of Education, Science and Technological Development (no. 200110, BS, NJ-Ž), British Heart Foundation (RG/11/28714, DM; FS/12/5/29339, DM), and BBSRC (BB/J005452/1, DM).

## Conflict of Interest

The authors declare that the research was conducted in the absence of any commercial or financial relationships that could be construed as a potential conflict of interest.

## Publisher’s Note

All claims expressed in this article are solely those of the authors and do not necessarily represent those of their affiliated organizations, or those of the publisher, the editors and the reviewers. Any product that may be evaluated in this article, or claim that may be made by its manufacturer, is not guaranteed or endorsed by the publisher.

## Abbreviations

ACTH: Adrenocorticotropic hormone
ADH: Antidiuretic hormone
ANP: Atrial natriuretic peptide
AP: Area postrema
AQP-2: Aquaporin 2
AT1R: Angiotensin II receptor type 1
AV3V: Anteroventral third ventricle
BHR: Borderline hypertensive rats
BPV: BP variability
BP: Blood pressure
BRS: Baroreflex sensitivity
CRH: Corticotropin-releasing hormone
CRHR2: corticotropin-releasing hormone receptor
DMV: dorsal motor nucleus of vagus
GABA: γ aminobutyric acid
GPCRs: G protein-coupled receptors
HF: High frequency
HPA: Hypothalamo–hypophyseal axis
HRV: Heart rate variability
IML: Intermediolateral nucleus
LF: Low frequency
MCNs: Magnocellular neurons
MnPO: Medial preoptic nucleus
NO: Nitrogen oxide
NTS: Nucleus tractus solitarius
OT: Oxytocin
OTR: Oxytocin receptor
OVLT: Organum vasculosum laminae terminalis
PCNs: Parvocelular neuron
PVN: Paraventricular nucleus
RVLM: Rostral ventrolateral medulla
SFO: Circumventricular subfornical organ
SHR: Spontaneously hypertensive rats
SND: Sympathetic nerve discharge
SPANs: Spinally projecting pre-utonimic neurons
VLF: Very low frequency
VLM: Ventrolateral medulla
VP: Vasopressin
VR: Vasopressin receptor
